# Relationship between Trace Element in Tumor and Prognosis in Lung Cancer Patients

**DOI:** 10.3390/medicina57030209

**Published:** 2021-02-26

**Authors:** Hirotaka Saikawa, Hiromi Nagashima, Katsuya Cho, Ryosuke Chiba, Koichiro Sera, Wataru Shigeeda, Makoto Tomoyasu, Hiroyuki Deguchi, Fumiaki Takahashi, Hajime Saito, Tamotsu Sugai, Makoto Maemondo

**Affiliations:** 1 Division of Pulmonary Medicine, Department of Internal Medicine, Iwate Medical University School of Medicine Yahaba, Iwate 028-3609, Japan; elysion-saikawa@hotmail.co.jp (H.S.); all-checker1983@m7.dion.ne.jp (H.N.); m11061kc@yahoo.co.jp (K.C.); imu_fc@yahoo.co.jp (R.C.); 2 Cyclotron Research Center, Iwate Medical University School of Medicine Takizawa, Iwate 020-0603, Japan; ksera@iwate-med.ac.jp; 3 Department of Thoracic Surgery, Iwate Medical University School of Medicine Yahaba, Iwate 028-3609, Japan; shigeeda@iwate-med.ac.jp (W.S.); tomoyasu@iwate-med.ac.jp (M.T.); hdeguchi@iwate-med.ac.jp (H.D.); hasaito@iwate-med.ac.jp (H.S.); 4 Department of Information Science, Iwate Medical University, Yahaba, Iwate 028-3609, Japan; ftakahas@iwate-med.ac.jp; 5 Department of Molecular Diagnostic Pathology, Iwate Medical University School of Medicine Yahaba, Iwate 028-3609, Japan; tsugai@iwate-med.ac.jp

**Keywords:** elemental analysis, particle-induced X-ray emission (PIXE), respiratory disease, lung cancer, chromium, Cr, cobalt, Co

## Abstract

*Background and Objectives:* This study aimed to observe the relationship between trace element concentrations in lung tissue from lung non-small cell lung carcinoma (NSCLC) patients and prognosis. *Materials and Methods:* The concentrations of various trace elements in the lung tissues were measured by a particle-induced X-ray emission (PIXE) system, and the results were analyzed for statistical significance. Eight essential trace elements, Cr, Mn, Fe, Co, Cu, Zn, Se, and Mo, were analyzed. We investigated the relationship between trace element concentrations and disease-free survival (DFS) and overall survival (OS) in NSCLC patients. *Results:* A total of 129 NSCLC patients and 20 control patients were included in this study. As for DFS, Co was the only element that showed a significant difference, and the high Co group had better DFS (HR: 0.352, 95% CI = 0.128–0.97). No significant difference was observed for Cr, Mn, Fe, Se, or Mo, but DFS tended to be better in the high trace element group. No significant difference was observed for Cu and Zn, but DFS tended to be good in the low trace element group. As for OS, Cr was the only element that showed a significant difference, and the high Cr element group had better OS (HR: 0.477, 95% CI = 0.128–0.97). *Conclusions:* This study suggests that the prognosis is good in lung cancer cases with high intratumoral concentrations of Co and Cr. The dynamics of trace elements in body and in tumor tissue have not been well established, and we consider that more research is necessary in the future.

## 1. Introduction

Lung cancer is one of the most common cancers. Although molecular targeting drugs and immune checkpoint inhibitors have been developed recently, mortality of lung cancer remains high [1]. To overcome this, it is important to find prognostic factors as well as develop new agents for treating lung cancer. [2]. Known prognostic factors include age, performance status, smoking status, and clinical stage.

We focused on the relationship between trace elements in lung cancer and prognosis. Trace elements are required in minute quantities for an organism to maintain proper physical functioning. Among them, elements essential for human life are called “essential trace elements”. Essential trace elements include Cr, Mn, Fe, Co, Cu, Zn, Se, Mo, and I. In our previous study, we analyzed the following trace elements by particle-induced X-ray emission (PIXE): Fe, Co, Ni, Cu, Zn, Ga, Ge, As, Se, Br, Rb, Sr, Y, Zr, Nb, Mo, Pd, Ag, Cd, In, Sn, Sb, Te, I, Cs, Ba, Ce, W, Pt, Au, Hg, and Pb. Pd, Ag, Cd, In, Sn, Sb, Te, I, Cs, Ba, Ce, W, Pt, Au, Hg, and Pb were not detected in any of the 129 non-small cell lung carcinoma (NSCLC) patients. However, Fe, Zn, and Br were detected in all 129 NSCLC patients.

This previous study showed that trace elements in lung cancer tissue and normal lung tissue are related to lung adenocarcinoma cancer (LADC) in both smokers and never-smokers. The concentrations of Fe, Co, Ni, Cu, Zn, and Br were significantly higher in the KRAS mutation and wild-type groups of LADC than in the nonmalignant control group, regardless of whether the samples were from tumor or normal lung tissues. The concentrations of these six trace elements were significantly higher in smokers than in nonsmokers [3]. The study showed significant differences in the concentrations of six trace elements, including Fe, Co, Ni, Cu, Zn, and Br, between tissues from LADC groups and the nonmalignant control group. This previous study used particle-induced X-ray emission (PIXE) to detect trace elements in surgically resected NSCLC tissues.

PIXE analysis has been used in the investigation of the elemental nature of complicated structures, such as human tissue sections, and provides elemental composition and trace concentrations for most elements with high sensitivity. We reported that PIXE analysis is a powerful tool for the elemental analysis of tissue samples [4,5,6]. In the clinical setting, however, it remains to be established whether these trace elements can be considered as prognostic factors.

In the current study, we evaluated the relationship between essential trace elements and disease-free survival (DFS) and overall survival (OS) in tumor tissues of NSCLC patients.

## 2. Materials and Methods

### 2.1. Method of the Current Study

Based on the results of our previous study, we analyzed 8 essential trace elements: Cr, Mn, Fe, Co, Cu, Zn, Se, and Mo. The essential trace element I was not detected in any patient and was thus excluded. Of the 150 patients involved in our previous study, 129 were NSCLC patients and 20 were control patients, who underwent surgery for benign pulmonary disease including pneumothorax. Characteristics of the control patients were shown in our previous paper reported by Chiba et al. [3]. The cut-off value was the average of the detection values of Cr, Mn, Fe, Co, Cu, Zn, Se, and Mo in the 20 control patients. Of the NSCLC patients, DFS and OS were analyzed by dividing patients into those above the cut-off value group (high trace element group) and those below the cut-off value group (low trace element group). We defined OS as the period from the date of the first visit to our hospital to the last confirmed date of survival or death, and DFS was the period from the date of surgery to the first date of recurrence of lung cancer, occurrence of other cancers, or death. OS and DFS were examined from the data in the Cancer Registry Room and the electronic medical records in our hospital.

### 2.2. Patient Population

Between January 2013 and December 2016, 1157 patients who had undergone surgical resection at Iwate Medical University Hospital were screened. Among them, 130 patients with NSCLC and 20 control patients who could be analyzed for trace elements were identified. In our previous study, all primary diagnoses were reviewed by experienced pathologists at each site according to the World Health Organization (WHO) nomenclature for adenocarcinomas. Specimens chosen for analysis were from patients with a confirmed diagnosis of NSCLC and tumors measuring greater than 20 mm, and specimens in which the percentage of tumor cells relative to other cells (e.g., stromal cells, pre-existing epithelial cells, and inflammatory infiltrate) were estimated to be >70% by pathologists were selected for analyses. Data collected in each case included age, sex, smoking history, and tumor-node-metastasis (TNM) stage. Formalin-fixed paraffin-embedded (FFPE) clinical tissue blocks were collected for mutation analyses of EGFR and KRAS. Based on hematoxylin and eosin staining, an additional block with less than 5% tumor cellularity from the same patient diagnosed with a tumor was used as the normal lung tissue sample. The study was conducted according to the provisions of the Helsinki Declaration and approved by the Ethics Committee at Iwate Medical University School of Medicine (H26–70), and all participants signed written consent forms before participating in the study. 

### 2.3. Mutational Status Analysis

Tumor samples from enrolled patients whose samples were available were obtained as FFPE samples in 5-µm-thick sections on glass slides. For EGFR gene mutation analyses, the tissues were removed from the slides by scraping, and DNA was extracted. The EGFR gene status was analyzed using the PNA-LNA PCR clamp method for exon 18 G719X, exon 20 T790M, and exon 21 L858R/L861Q and the fragment method was used for exon 19 deletion (added to the PNA-LNA PCR clamp method) [7]. 

### 2.4. PIXE Analysis

Untreated organ samples weighing less than 50 µg were placed on a backing film (4-µm-thick Proline film) and fixed with 1% collodion solution, and the samples were quantitatively analyzed based on the standard-free method for untreated organs. A 2.9 MeV proton beam extracted from a cyclotron at Nishina Memorial Cyclotron Center (NMCC) was transported to a PIXE vacuum chamber in a PIXE room and irradiated the targets. Emitted X-rays were simultaneously measured with two Si (Li) detectors. X-ray absorbers (300-µm-thick Mylar films) were used for the No. 1 detector, while an X-ray collimator consisting of graphite with an aperture of 1.5 mm in diameter was placed directly in front of detector No. 2 to adjust the counting rate of the X-rays. The typical beam current in the vacuum chamber was approximately 70 nA, and the spot size of the beam was 5 mm in diameter. A more detailed description of the arrangement is provided in a previous publication [8]. In this study, we tracked and analyzed the data from the previous study.

### 2.5. Statistics and Analysis

OS and DFS were statistically analyzed using Cox regression with SAS Ver9.4 (SAS institute inc., Cary, NC, USA). The covariate-adjusted survival curve was estimated by the method proposed by Makuch [9]. In DFS and OS of this study, age (under 75 years vs. 75 years or older), gender (male vs. female), smoking status (none vs. current/former), staging (stage I vs. other), histological type (adenocarcinoma vs. squamous cell carcinoma) and EGFR mutation (positive vs. negative) were adjusted as covariates.

## 3. Results

### 3.1. Patients

**Figure 1:**
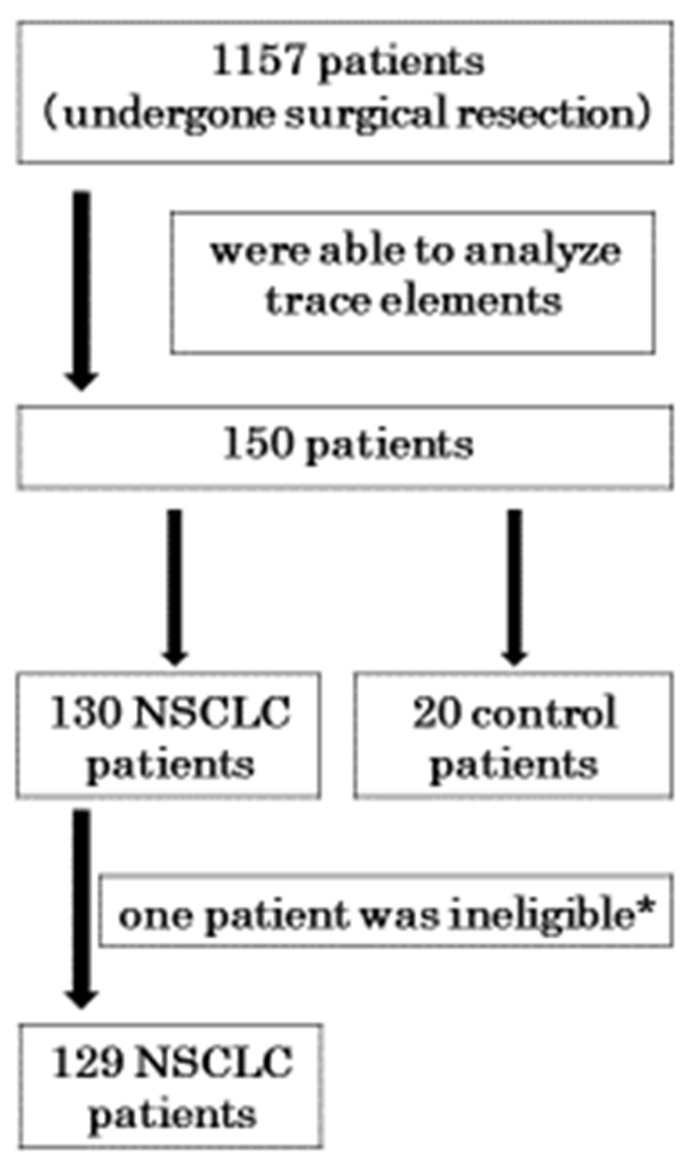
This shows the patients involved in this study; 129 non-small cell lung carcinoma (NSCLC) patients and 20 control patients participated. * This patient could not be followed to obtain data of disease-free survival (DFS) and overall survival (OS).

Figure 1 shows the patients who participated in this study. Of the 1157 patients who underwent surgical resection of lung tissue at our hospital, 150 whose trace elements could be analyzed participated in the study. They included 130 NSCLC patients and 20 control patients. Of the 130 NSCLC patients, one was unable to be followed after surgical resection and was excluded from the study.

### 3.2. Characteristics

Table 1 and Table 2 show the characteristics of the 129 NSCLC patients divided into a high trace element group and a low trace element group according to eight trace elements. The average trace element concentrations of the 20 control patients were Cr 2.8, Mn 0.221, Fe 182.16, Co 0.04, Cu 1.82, Zn 1.66, Se 0.19, and Mo 0.64. Based on these values, the patients were divided into a high trace element group and low trace element group. The number and proportion in the high trace element group for each trace element were as follows: Cr: 92 (71%), Mn: 58 (45%), Fe: 93 (72%), Co: 79 (61%), Cu: 28 (22%), and Zn: 102 (78%), Se: 65 (50%), and Mo: 56 (43%). Table 1 and Table 2 show the age, gender, smoking status, pathological stage, histology, and EGFR mutation of the two groups.

### 3.3. Disease-Free Survival (DFS)

**Figure 2:**
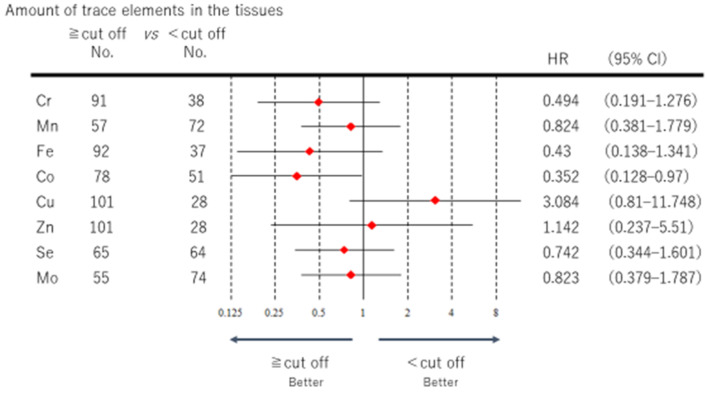
The DFS of each of the eight essential trace elements is represented by a forest plot, and the hazard ratio (confidence interval) is shown in the right column. The closer the range is to 0, the better the prognosis in the high trace element group vis-à-vis the low trace element group.

Figure 2 shows a forest plot of DFS for each of the trace elements. Co was the only element that showed a significant difference. The low Co group showed a significantly poorer DFS than the high Co group the hazard ratio (HR) of Co was 0.352 and the 95% confidence interval (95% CI) was 0.128–0.97), the high trace element group having better DFS. The HR (95% CI) of each trace element was as follows: Cr 0.494 (0.191–1.276), Mn 0.824 (0.381–1.779), Fe 0.43 (0.138–1.779), Se 0.742 (0.344–1.601), and Mo 0.823 (0.379–1.787). No significant difference was observed for Cr, Mn, Fe, Se, or Mo, but DFS tended to be better in the high trace element group. The HR of Cu was 3.084, with a 95% CI of 0.81–11.748, and the HR of Zn was 1.142, with a 95% CI of 0.237–5.51. No significant difference was observed for Cu or Zn, but DFS tended to be better in the low trace element group.

**Figure 3:**
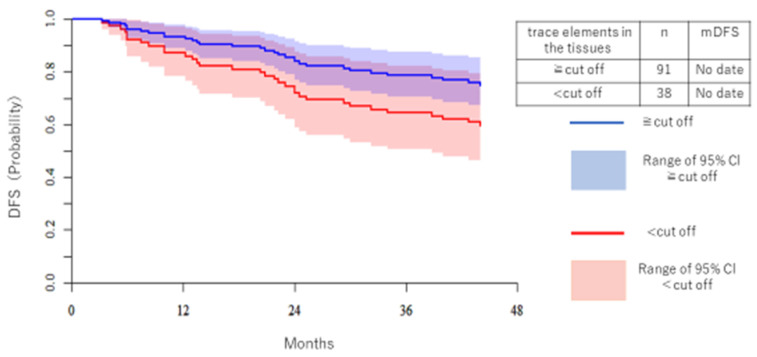
The DFS of Cr is shown separately for the high trace element group and the low trace element group. The vertical axis is the patient ratio, and the horizontal axis is the period (months). The blue line is the high trace element group and the red line is the low trace element group. The median DFS was immature.

Figure 3 shows DFS and the Kaplan–Meier curve (KM) of Cr. No significant difference was observed, but DFS tended to be better in the high trace element group. Median DFS (mDFS) was immature.

**Figure 4:**
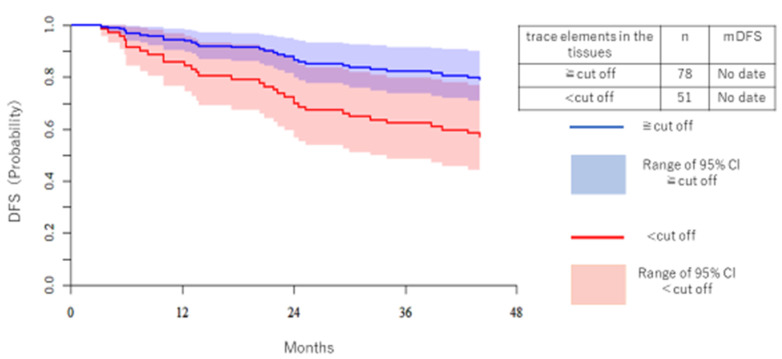
The DFS of Co is shown separately for the high trace element group and the low trace element group. The vertical axis is the patient ratio, and the horizontal axis is the period (months). The blue line is the high trace element group and the red line is the low trace element group. The median DFS was immature.

Figure 4 shows the DFS and KM of Co. The high trace element group had better DFS. mDFS was immature.

### 3.4. Overall Survival (OS)

**Figure 5:**
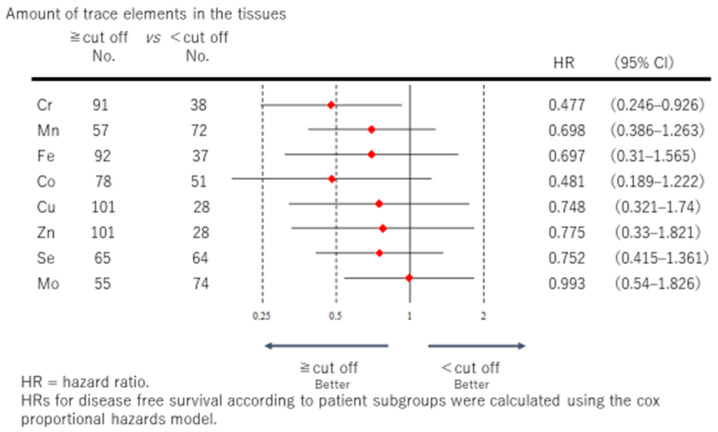
The OS of each of the eight essential trace elements is represented by a forest plot, and the hazard ratio (confidence interval) is shown in the right column. The closer the range is to 0, the better the prognosis in the high trace element group vis-à-vis the low trace element group.

Figure 5 shows a forest plot of OS related to each trace element. Cr was the only element that showed a significant difference. The low Cr group showed a significantly poorer OS than the high Cr group (HR: 0.477, 95% CI = 0.128–0.97), the high trace element group having better OS. The HR (95% CI) of each trace element was as follows: Mn 0.698 (0.386–1.263), Fe 0.697 (0.31–1.74), Co 0.481 (0.189–1.222), Cu 0.748 (0.321–1.74), Zn 0.775 (0.33–1.821), Se 0.752 (0.415–1.361), and Mo 0.993 (0.54–1.826). No significant difference was observed for any other elements, but OS tended to be higher in the high trace element group.

**Figure 6:**
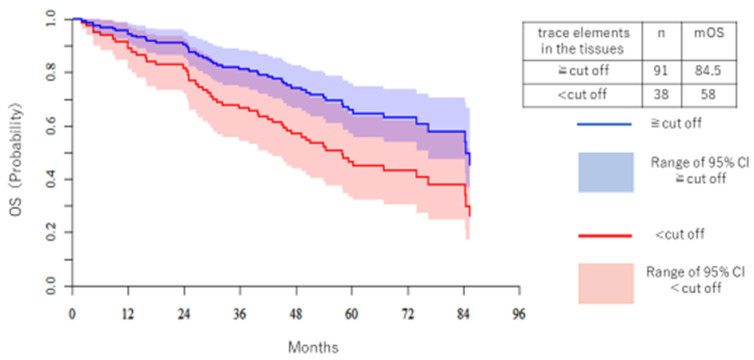
The OS of Cr is shown separately for the high trace element group and the low trace element group. The vertical axis is the patient ratio, and the horizontal axis is the period (months). The blue line is the high trace element group and the red line is the low trace element group. The median OS was 84.5 months in the high trace element group and 58 months in the low trace element group.

Figure 6 shows the DFS and KM of Cr. The high trace element group had better OS. Median OS (mOS) of the high Cr group was 84.5 months, and for the low Cr group it was 58 months.

**Figure 7:**
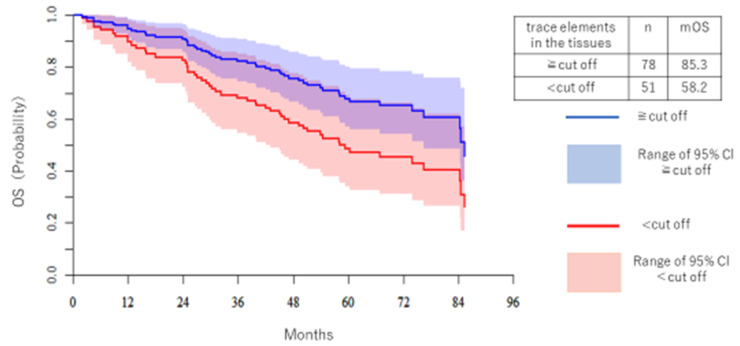
The OS of Co is shown separately for the high trace element group and the low trace element group. The vertical axis is the patient ratio, and the horizontal axis is the period (months). The blue line is the high trace element group and the red line is the low trace element group. The median OS was 85.3 months in the high trace element group and 58.2 months in the low trace element group.

Figure 7 shows OS and KM of Co. No significant difference was observed, but OS tended to be higher in the high trace element group. mOS of the high Co group was 85.3 months, and for the low Co group it was 58.2 months.

### 3.5. Correlation between Tumor Tissue and Nontumor Tissue

**Figure 8:**
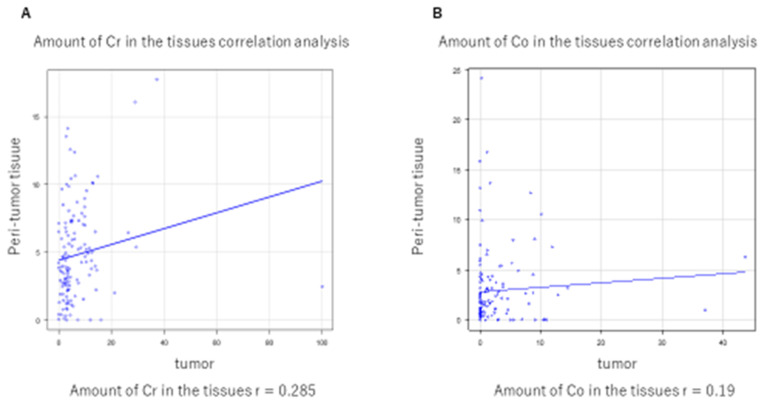
(**A**) shows that the correlation coefficient (r) of Cr was 0.285. (**B**) shows that the correlation coefficient (r) of Co was 0.19.

The correlation between the trace element concentrations in the tumor tissue and the concentrations in the nontumor tissue (normal lung tissue) in the obtained sample was examined by the Spearman’s rank correlation coefficient. In one patient, Cr was significantly different from the average value, so this was excluded from the analysis. Figure 8A shows that the correlation coefficient of Cr was 0.285. Figure 8B shows that the correlation coefficient of Co was 0.19. There was no correlation in Co and Cr concentration between tumor tissue and peritumoral normal tissue in the identical specimens.

## 4. Discussion

This study focused on the essential trace elements of Cr, Mn, Fe, Co, Cu, Zn, Se, and Mo in lung tumors and analyzed the relationship between each trace element and postoperative DFS and OS. High Cr and Co in lung tumors were associated with a good prognosis after surgery. Patients with high Cr and Co in the tumor showed statistically significant prolongation of DFS and OS, respectively. This result is a new finding for the association between trace elements and operable lung cancer.

In our previous study, we demonstrated significant differences in the concentrations of six trace elements (Fe, Co, Ni, Cu, Zn, and Br) between lung adenocarcinoma patients and control patients. These six trace elements have the potential to promote carcinogenesis and cancer progression [3]. Although previous reports focused on carcinogenesis, this study spotlighted the outcome of surgery and essential trace elements.

Chromium is one of the essential trace elements, and the main form found in the body is trivalent chromium (Cr3 +), which is found in many foods. This form of chromium is thought to be involved in maintaining normal insulin function, lowering blood glucose and maintaining normal glucose metabolism. In addition, trivalent Cr is an important element that has been considered to play a role as a component of proteolytic enzymes and a component of saving serum cholesterol homeostasis. This element is also present in other forms such as hexavalent (Cr6 +). However, Cr6 +, unlike Cr3 +, is toxic and is caused by factory pollution. It has been epidemiologically pointed out that Cr is associated with carcinogenesis of lung cancer. It was reported that the incidence of lung cancer was high in workers dealing with hexavalent Cr [10].

It is impossible to distinguish between hexavalent Cr and trivalent Cr by PIXE. The risk of exposure to hexavalent Cr is not so high in ordinary patients not dealing with hexavalent Cr and hexavalent Cr (VI) can also be reduced to the trivalent Cr(III) in the epithelial lining fluid of the lungs by ascorbate and glutathione [11,12]. Consequently, we considered trivalent Cr to be the main component in this study.

To date, there has been no report of an association between the Cr concentration in the tumor tissue of lung cancer and prognosis after surgery. This study reported that a higher concentration of Cr in the tumor was associated with better DFS and OS. This result is contrary to lung toxicity induced by inhalation of hexavalent Cr. As mentioned above, we found no correlation between the trace element concentrations of tumor tissue and nontumor tissue. We do not consider this result to be derived from inhalation of elements. To elucidate this phenomenon, further clinical and in vitro studies are needed.

It is generally reported that alloys of Co and tungsten carbide are likely to be carcinogenic to the human body, including to the lungs. Co, as an essential trace element in the body, is a constituent of Vitamin B12, and is involved in hematopoiesis, nerve protection, and coenzymes [13]. We could not find any studies evaluating Co concentration in the tumor tissue of lung cancers. Although the higher concentration of Co in tumor tissue was shown in our previous study, the high Co group had a significantly better DFS than the low Co group in the current study (3). There was no correlation between Co concentration in the tumor tissue and the trace element concentration in the nontumor tissue, and likewise for Cr. It was unclear whether the high concentration of Co in lung tumor tissue was derived from intake or implanted metal.

In this study, we were not able to evaluate carcinogenesis of essential trace elements or evaluate the mechanisms of association between prognosis and trace elements. These differences of OS among essential elements may be associated with factors that are not directly related to carcinogenesis. It is unclear why prognosis is better when the concentrations of Cr and Co in the tumor tissue are high. Although it is a different trace element, it has been reported that aluminum inhibits tumor growth by preventing binding to carcinogens [14]. It is possible that Co and Cr also suppress tumor growth by mechanisms yet to be elucidated. We consider the DFS and OS in this study might have several limitations because it is difficult to obtain all the factors related to DFS and OS in a retrospective study. In addition, although it is pointed out that there may be differences in trace element concentrations between FFPE tissue and non-FFPE tissue like frozen section, we only investigated FFPE tissues and did not compare non-FFPE tissues [15,16].

## 5. Conclusions

The results of this study suggest that prognosis is better in lung cancer patients with high intratumoral concentrations of Co and Cr. The dynamics of trace elements in body and in tumor tissue have not been well established. The relationship between trace elements and environmental factors, carcinogenicity, and accumulation in the body is not sufficiently elucidated. We hope that this study will give an opportunity to spotlight the relationship between trace elements and recurrence and progression of lung tumors. More research of this field is warranted.

## Figures and Tables

**Table 1:** This shows the characteristics of 129 NSCLC patients divided into a high trace element group and a low trace element group for Cr, Mn, Fe, and Co.

	Cr (High)	Cr (Low)	Mn (High)	Mn (Low)	Fe (High)	Fe (Low)	Co (High)	Co (Low)
N = 129	91	38	57	72	92	37	79	50
Age								
75≤	33 (36%)	10 (26%)	19 (33%)	24 (33%)	31 (34%)	12 (32%)	23 (29%)	20 (40%)
<75	58 (64%)	28 (74%)	38 (67%)	48 (67%)	61 (66%)	25 (68%)	56 (71%)	30 (60%)
Sex								
Male	59 (64%)	19 (50%)	43 (75%)	35 (49%)	58 (63%)	20 (54%)	52 (66%)	26 (52%)
Female	32 (36%)	19 (50%)	14 (35%)	37 (51%)	44 (37%)	17 (46%)	27 (34%)	24 (48%)
Smoking status								
Current or former	54 (59%)	14 (37%)	36 (63%)	32 (44%)	53 (58%)	15 (41%)	51 (65%)	17 (34%)
Never	37 (41%)	24 (64%)	21 (37%)	40 (56%)	39 (42%)	22 (59%)	28 (35%)	33 (66%)
Staging								
II≤	24 (26%)	11 (29%)	12 (21%)	23 (32%)	21 (23%)	14 (38%)	20 (25%)	15 (30%)
I	67 (74%)	27 (71%)	45 (79%)	49 (68%)	71 (77%)	23 (62%)	59 (75%)	35 (70%)
Histologic features								
Adeno	83 (91%)	27 (71%)	51 (89%)	59 (82%)	89 (97%)	11 (30%)	76 (96%)	34 (68%)
Squamous	8 (9%)	11 (29%)	6 (11%)	13 (18%)	3 (3%)	16 (70%)	3 (4%)	16 (32%)
EGFR mutation								
(+)	7 (8%)	13 (34%)	7 (12%)	13 (18%)	14 (15%)	6 (16%)	5 (6%)	15 (30%)
(−)	84 (92%)	25 (66%)	50 (88%)	59 (82%)	78 (85%)	31 (84%)	74 (94%)	35 (70%)

**Table 2:** This shows the characteristics of 129 NSCLC patients divided into a high trace element group and a low trace element group of Cu, Zn, Se, and Mo.

	Cu (High)	Cu (Low)	Zn (High)	Zn (Low)	Se (High)	Se (Low)	Mo (High)	Mo (Low)
N = 129	101	28	101	28	64	65	56	73
Age								
75≤	35 (35%)	8 (32%)	31 (31%)	12 (43%)	22 (34%)	21 (32%)	19 (34%)	24 (33%)
<75	66 (65%)	20 (68%)	70 (69%)	16 (57%)	42 (66%)	44 (68%)	37 (66%)	49 (67%)
Sex								
Male	61 (60%)	17 (61%)	59 (58%)	19 (68%)	43 (67%)	35 (54%)	33 (59%)	45 (62%)
Female	40 (40%)	11 (39%)	42 (42%)	9 (32%)	21 (33%)	30 (46%)	23 (41%)	28 (38%)
Smoking status								
Current or former	52 (51%)	16 (57%)	52 (51%)	16 (57%)	37 (58%)	31 (48%)	33 (59%)	35 (48%)
Never	49 (49%)	12 (43%)	49 (49%)	12 (43%)	27 (42%)	34 (52%)	23 (41%)	38 (52%)
Staging								
II≤	28 (28%)	7 (25%)	26 (26%)	9 (32%)	14 (22%)	21 (32%)	13 (23%)	22 (30%)
I	73 (72%)	21 (75%)	75 (74%)	19 (68%)	50 (78%)	44 (68%)	43 (77%)	51 (70%)
Histologic features								
Adeno	97 (96%)	13 (46%)	99 (98%)	11 (39%)	59 (92%)	51 (78%)	46 (82%)	64 (88%)
Squamous	4 (4%)	15 (54%)	2 (2%)	17 (61%)	5 (8%)	14 (22%)	10 (8%)	9 (12%)
EGFR mutation								
(+)	19 (19%)	1 (4%)	14 (14%)	6 (21%)	6 (9%)	14 (22%)	5 (9%)	15 (21%)
(−)	82 (81%)	27 (96%)	87 (86%)	22 (79%)	58 (91%)	51 (78%)	51 (91%)	58 (79%)
